# Lumbopelvic dysfunction and depression in pregnant women: a cross-sectional study

**DOI:** 10.1186/s12884-025-08287-4

**Published:** 2025-11-28

**Authors:** Veronica Isabel Mejía-Mercado, Asunción Ferri-Morales, Vicente Martínez-Vizcaíno, María Esther Suárez-García, Cristina Gallego-Gómez, Claudia Andrea Quezada-Bascuñán, Eva Rodríguez-Gutiérrez, Celia Ramírez-Luque, Ana Torres-Costoso

**Affiliations:** 1 Unit of Pelvic Floor Physiotherapy, Dr. Alejandro Dávila Bolaños Military Teaching Hospital, Managua, Nicaragua; 2 Faculty of Physiotherapy and Nursing, University of Castilla-La Mancha, Toledo, Spain; 3 Health and Social Research Center, University of Castilla-La Mancha, Cuenca, Spain; 4 Facultad de Ciencias de la Salud, Universidad Autónoma de Chile, Talca, Chile; 5 Department of Gynecology and Obstetrics, Dr. Alejandro Dávila Bolaños Military Teaching Hospital, Managua, Nicaragua; 6 Unit of Physiotherapy, Health Center of Camarena, Toledo, Spain

**Keywords:** Pregnancy, Urinary incontinence, Fecal incontinence, Constipation, Sexual dysfunction, Lumbopelvic pain, Depression

## Abstract

**Background:**

Depression and lumbopelvic dysfunction, including low back pain and pelvic floor disorders, are both common conditions that affect women during pregnancy; however, there is a gap in understanding the association between the two. Thus, the aim of this study was to identify lumbopelvic risk factors for depression in pregnant women.

**Methods:**

In this cross-sectional study, lumbopelvic dysfunction (low back pain, urinary incontinence, fecal incontinence, constipation, and/or sexual dysfunction) was examined in 375 pregnant Nicaraguan women. The women were categorized into two groups, pregnant women with and without symptoms of depression, based on a cut-off of ≥ 13 on the Edinburg Depression Scale, to define higher symptoms of depression, and ANOVA models were applied to assess the role of sociodemographic data and lumbopelvic dysfunction on depression risk. Additionally, logistic regression analyses were conducted to identify lumbopelvic factors independently associated with a positive status of depression.

**Results:**

Pregnant women with depression symptoms presented significantly worse lumbopelvic function than did their peers without symptoms of depression. Depression status was more strongly associated with low back pain (OR 2.17; IC 1.22, 3.86), constipation (OR 2.83; IC 1.52, 5.26) and sexual dysfunction (OR 1.99; IC 1.17, 3.40) but not in those with urinary incontinence (OR 1.50; IC 0.85, 2.65) or fecal incontinence (OR 1.70; IC 0.74, 3.91).

**Conclusion:**

The association between depression during pregnancy and lumbopelvic dysfunction was significant, mainly in the presence of low back pain, constipation, and sexual dysfunction. These findings may be clinically useful for depression risk screening and for guiding future interventions in pregnant women, although their impact should be confirmed by longitudinal studies to understand the long-term consequences for both mothers and babies.

**Supplementary Information:**

The online version contains supplementary material available at 10.1186/s12884-025-08287-4.

## Introduction

The perinatal period (from antenatal to 12 months postpartum) represents a critical period for maternal mental health due to hormonal, emotional, and social changes [1]. These changes can trigger episodes of depression with a greater risk of adverse maternal and child outcomes [2]. In addition, the impact is disproportionately greater in those living in low- and middle-income countries, with a prevalence affecting one in four women [3], probably due to the increased prevalence of risk factors such as HIV, intimate partner violence, and war and conflict combined with limited treatment options in these regions [4].

Historically, greater attention has been given to postpartum depression and its associated risk factors; however, despite depression being a common medical and psychological condition during pregnancy, there remains a lack of focus on mental health issues [5]. This major life event may represent a period of increased vulnerability to the onset or recurrence of depression; in fact, some women experience their first depressive episode during this time [6].

During pregnancy, physiological, hormonal and postural changes can compromise the stability of the back and pelvic floor muscles, often leading to lumbopelvic dysfunction, a multifactorial disorder of the lumbopelvic region that includes conditions as low back pain or pelvic floor disorders. which may involve impaired motor control, muscle imbalances, and pain that affects posture, movement, and functional capacity [7]. Approximately three-fifths of pregnant women usually experience low back pain in the second trimester of pregnancy [8], which is associated with increased physical morbidity and disability, as well as psychological distress in the postpartum period [9]. Pelvic floor disorders such as urinary incontinence, fecal incontinence, constipation, and sexual dysfunction are also highly prevalent and often underreported [10]. The impact and negative effects on the health and quality of life of pregnant women are significant, with important consequences for physical and mental well-being, including substantial functional limitations, embarrassment, social isolation, problems with activities of daily living, negative impacts on personal relationships, and difficulties engaging in leisure and recreational activities [11].

The prevalence of anxiety and depression in women with lumbopelvic disorders is high, particularly among those with IU, FI, and organ prolapse [12]. These conditions also occur during the postpartum period [13]. However, and despite the plausibility of the relationship between antenatal depression and lumbopelvic disorders during pregnancy, existing research has approached these conditions in isolation. Most studies addressing low back pain or pelvic floor disorders have focused on physical and functional outcomes, with limited exploration of their emotional or psychological consequences, even when both conditions may share overlapping pathways of vulnerability, such as sleep disturbance, reduced mobility, loss of independence, and a diminished self-image [14–17]. Thus, a dual-perspective approach to exploring these conditions seems important.

In addition, early identification of women at high risk of depression during pregnancy is important for its prevention and subsequent maternal and fetal outcomes. Numerous sociodemographic, obstetric, and psychological factors have been associated with this problem [18]. However, despite extensive research on lumbopelvic dysfunctions in pregnant women, there is still limited understanding of how these physical symptoms may influence mental health, particularly in relation to depression during pregnancy. In addition, exploring the association could help to promote multidisciplinary approaches, improving maternal well-being through more holistic and integrated care, according to individual needs. Therefore, the present study aimed to estimate the potential relationships between low back pain, urinary incontinence, fecal incontinence, constipation, sexual function, and depressive symptoms in pregnant women, even after adjusting for relevant anthropometric and sociodemographic variables.

## Methods

### Study design and participants

This study is a cross-sectional analysis based on baseline data from a prospective study aimed at assessing the incidence of adverse outcomes in women with lumbopelvic dysfunction during pregnancy. Between May and October 2024, 375 pregnant women in their second trimester were consecutively recruited at the Gynaecology and Obstetrics Service of Dr. Alejandro Dávila Bolaños Military School Hospital, Managua (Nicaragua).

The inclusion criteria consisted of healthy women over 18 years of age with a normal second trimester pregnancy who provided informed consent. The exclusion criteria were high-risk obstetric pregnancies, fetal malformations, previous back, abdominal or urogenital surgery or difficulties in completing the questionnaires.

This study was conducted in accordance with the Declaration of Helsinki, ensuring participants’ privacy and confidentiality. Ethical approval was obtained from the hospital’s ethics committee prior to data collection (reference: SCEHM-2024-10). The study’s objectives and procedures were explained in detail, and written informed consent was obtained from all participants as a prerequisite for inclusion. In addition, the study adhered to the STROBE guidelines for reporting observational research (Table S1).

### Anthropometric and sociodemographic data

At the first antenatal visit, weight and height were collected. Weight was measured twice with the pregnant woman barefoot and in light clothing. Height was also measured twice with the pregnant woman barefoot, upright, and with the sagittal midline touching the back board, both measurements were performed using the HT485 device (Graham-Field Health Products). Body mass index (BMI) was calculated as weight in kg divided by the square of the height in meters and categorized as normal weight, < 25 kg/m2; overweight, 25–30 kg/m2; and obesity, >30 kg/m2 [19]. Age, parity (nulliparous/multiparous), history of mental illness, family income and educational level were also assessed.

Family income level was categorized as low (first quartile), medium (second and third quartiles), or high (fourth quartile) following the classification previously used [20].

The educational level of the pregnant women was considered as: [1] no literacy [2], no studies [3], elementary studies [4], secondary studies [5], high school, and [6] university studies. These 6 categories were collapsed into 3 categories: primary education, including functionally illiterate mothers with no formal education or those who had not completed primary education (categories 1–3); secondary education, including complete primary or high school/secondary education (categories 4 and 5); and technical/university education, including technical degrees, university degrees, and master’s or doctoral degrees (category 6) [21].

### Depression condition

Depression was assessed using the Edinburgh Depression Scale (EDS), a reliable and validated instrument for screening depression during pregnancy [22]. This self-administered questionnaire consisting of 10 items with four response options. Scores range from 0 to 30, with higher scores indicating greater severity of depression [23]. This scale has been validated for detecting depression during pregnancy. An EDS cut-off score ≥ 11 maximizes the combined sensitivity and specificity, whereas a cut-off score ≥ 13, although less sensitive, is more specific and has been widely used to identify pregnant women with more severe depressive symptoms [24, 25], particularly during the second trimester of pregnancy [26]. In addition, a history of mental illness was recorded (e.g., anxiety, depression, etc.).

### Lumbopelvic dysfunction

#### Low back pain

Low back pain has been defined as pain localized to the lumbar spine with or without radiation to the hip or leg [27]. The severity of low back pain was assessed using a numerical pain rating scale (NPRS). It consists of a 10-point scale, with “0” representing “no pain” and “10” representing “most severe pain imaginable”, where women are asked to indicate the value of their pain on the scale at the time of assessment. A cut-off higher than 3 has been shown to predict moderate disability [28].

#### Urinary incontinence

To assess the presence of urinary incontinence and compute the relative score reflecting symptom severity, the reliable and validated Spanish version of the International Consultation Incontinence Questionnaire-Short Form (ICIQ-SF) was used [29]. The ICIQ-SF score ranges from 0 to 21 points. The score is the weighted sum of three questions about the frequency of leakage (5 points), the amount of leakage (6 points) and interference with activities of daily living (10 points). Higher values indicate more severe incontinence [30]. On the basis of this score, the following categories can be established: no incontinence or mild incontinence (1–5 points), moderate incontinence [6–12], severe incontinence (13–18 points), and very severe incontinence (19–21 points) [31].

#### Fecal incontinence

The reliable and validated Cleveland Clinic Florida Fecal Incontinence (CCFI) scale was used to assess fecal incontinence severity through a questionnaire evaluating five parameters (frequency of incontinence of solids, incontinence of liquids, incontinence of flatus, use of continence pads, and lifestyle alteration), each scored from 0 to 4 points based on frequency [32]. The total score ranges from 0 points (no incontinence) to 20 points (maximum incontinence) [33]. The cut-off value for determining severity and its impact on quality of life was 9, so a Wexner score < 9 is considered mild fecal incontinence, and a score ≥ 9 is considered moderate/severe fecal incontinence [34].

#### Constipation

To assess constipation severity, the Wexner Constipation Scoring System (WCSS) was used. This reliable and validated scoring system [32] is composed of 8 items (frequency of bowel movements, difficult or painful evacuation, completeness of evacuation, abdominal pain, time per attempt, assistance for defecation, number of unsuccessful attempts at evacuation in a 24-h period, and duration of constipation), with scores ranging from 0 points (no constipation) to 30 points (severe constipation) [35]. Based on the grading of the questionnaire, constipation can be considered mild (score of 1–5 points), moderate (score of 6–10 points), severe (score of 11–15 points), or very severe (16–30 points) [36].

#### Sexual function

To evaluate sexual function, the reliable and validated Female Sexual Function Index (FSFI) was used [37]. This multidimensional self-report instrument contains 19 items distributed across six subscales. Items 3–14 and 17–19 are scored from 0 to 5; items 1, 2, 15, and 16 are scored from 1 to 5. The total score ranges from 2 to 36 points, where higher scores indicate better sexual function and sexual dysfunction with scores ≤ 26.5 [38].

### Statistical analysis

Sample size was calculated to detect an odds ratio of 1.5 for the association between lumbopelvic dysfunction and depression, assuming a 60% prevalence of lumbopelvic dysfunction and 40% prevalence of depression. Using Hsieh’s formula adjusted for multiple predictors with a variance inflation factor accounting for moderate correlation between covariates (R² = 0.30) and applying 80% power with a two-sided significance level of α = 0.05, the required sample size was calculated to be 325 participants [39]. The final sample size was adjusted to 375 participants to account for an anticipated 15% dropout rate. All calculations were performed using established statistical methods for multivariable logistic regression in epidemiological studies [40].

Normality of the distribution of continuous variables was checked using both statistical (the Kolmogorov–Smirnov test) and graphical (normal probability plot) approaches. Baseline differences according to depression status (defined by an EDS cut-off ≥ 13) were examined using analysis of variance (ANOVA) for continuous variables and chi-square tests for categorical variables. These comparisons were also repeated using an alternative EDS cut-off of ≥ 11.

The relationships between EDS and other risk factors, including age, BMI, socioeconomic status (family income level and educational level of the pregnant woman), parity, low back pain, urinary incontinence, fecal incontinence, constipation and sexual function, were estimated using Pearson’s or Spearman’s correlation coefficients, as appropriate.

**Fig. 1:**
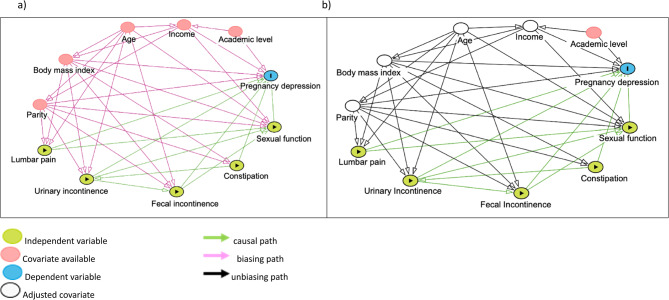
Directed acyclic graphs (DAG) for the causal structure of the relationships between pregnancy depression and the predictor factors studied. Panel (**a**) is the original DAG, Panel (**b**) is the DAG after adjustment for the minimum sufficient adjustment set for the overall effect (i.e., age, body mass index, income and parity)

To identify the minimally sufficient adjustment set (MSAS) for assessing the associations between depressive condition and anthropometric, sociodemographic and physical risk factors in pregnant women, we built a theoretical causal diagram based on previous associations between EDS and age, BMI, parity, family income level, academic level, low back pain and pelvic floor disorders available in the literature [12–41] (Fig. 1). A directed acyclic graph (DAG) was developed using the online tool DAGitty [42] to construct the covariates age, BMI, income, and parity, which were identified as the MSAS for pregnancy depression (Panel a). Panel b shows the DAG after adjustment for the covariates.

Logistic regression analyses were conducted to estimate the odds ratios and 95% confidence intervals for the status of depression (according to the abovementioned cut-off point of ≥ 13 in the EDS) across the categories of low back pain, urinary incontinence, fecal incontinence, constipation, and sexual function. For its categorization, we created dichotomous variables considering the “presence of low back pain, urinary incontinence, fecal incontinence and constipation” in women with moderate/severe severity, thus obtaining a NPRS score ≥ 3, ICIQ-SF score ≥ 5, CCFI score ≥ 9, and WCSS score ≥ 5 for each symptom and the “presence of sexual dysfunction” in women with a FSFI score ≤ 26.5. In addition, as it was identified by the MSAS, the analyses were also adjusted for age, BMI, family income level and parity. Additional analyses were also performed using a depression risk cut-off of ≥ 11 in the EDS.

All analyses were performed using the Statistical Package for the Social Sciences (SPSS, version 28.0; IBM Corp., Chicago) and Stata 17 software.

The level of significance was set at α = 0.05.

## Results

Table 1 displays the characteristics of the sample by depression status, defined as a score of ≥ 13 on the EDS. Among the 375 pregnant women included in the study, 84 (22.4%) showed symptoms of depression. There were no significant differences in sociodemographic characteristics (family income and educational level), obstetric history (primiparity versus multiparity), or pre-pregnancy history of mental illness (reported by 0.8% of participants) between those with and without depressive symptoms (all *P* > 0.05). All participants completed the study measurements.

**Table 1:** Characteristics of the study participants

	Pregnant women		
	Without symptoms of depression (*N*:291)	With symptoms of depression (*N*:84)	*p* value*
Age (years)	30.19 ± 4.82	29.00 ± 4.31	0.043
BMI (kg/m2)	22.37 ± 4.28	22.71 ± 4.64	0.526
Income level (cordobas)	15483.14 ± 9612.11	14157.14 ± 8277.51	0.252
Academic level, n(%)			0.012
Primary studies	4 (1.06%)	0	
Secundary studies	58 (15.40%)	30 (8.00%)	
Technical/University	229 (61.06%)	54 (14.40%)	
Parity, n (%)			0.114
Primiparous	125 (33.33%)	28 (7.46%)	
Multiparous	166 (44.26%)	56 (14.93%)	
History of mental illness, n(%)			0.065
No	290 (77.33%)	82 (21.86%)	
Yes	1 (0.26%)	2 (0.53%)	
EDS_Total Score	5.26 ± 3.95	16.85 ± 3.93	< 0.001
Low back pain (NPSR)	3.36 ± 2.75	5.42 ± 2.98	< 0.001
UI (ICIQ-SF)	2.71 ± 3.78	4.82 ± 4.72	< 0.001
FI (CCFI)	2.99 ± 2.81	4.18 ± 3.39	0.001
Constipation (WCSS)	5.91 ± 4.10	8.70 ± 3.89	< 0.001
Sexual Function (FSFI)	25.75 ± 7.19	22.66 ± 7.83	< 0.001

Values are means ± SD (quantitative variables) or n (%) (categorical variables). *Statistical significance, *p* ≤ 0.05

*Abbreviations*: *BMI* Body mass index, *CCFI* Cleveland Clinic Florida Fecal Incontinence, *EDS* Edinburgh Depression Scale, *FI* Fecal Incontinence, *FSFI* Female Sexual Function Index, *ICIQ-SF* International Consultation on Incontinence Questionnaire–Short Form, *NPRS* Numerical pain rating scale, *UI* Urinary Incontinence, *WCSS* Wexner Constipation Scoring System

Compared with those without symptoms, women with depressive symptoms had markedly higher scores on the depression scale (16.85 ± 3.93 vs. 5.26 ± 3.95, *p* < 0.001). Additionally, they also showed significantly greater impairment in health-related domains, as evidenced by higher scores for low back pain (NPRS: 5.42 [2.98] vs. 3.36 [2.75]; *P* < 0.001), urinary incontinence (ICIQ-SF: 4.82 [4.72] vs. 2.71 [3.78]; *P* < 0.001), fecal incontinence (CCFFI: 4.18 [3.39] vs. 2.99 [2.81]; *P* = 0.001), and constipation (WCSS: 8.70 [3.89] vs. 5.91 [4.10]; *P* < 0.001). Finally, sexual function was lower in the group with depressive symptoms (FSFI: 22.66 ± 7.83 vs. 25.75 ± 7.19, *p* < 0.001). Sensitivity analyses using a lower EDS cut-off (≥ 11) produced comparable results (see Table S2).

Bivariate correlation analyses (Table 2) indicated that depression was significantly associated with low back pain (*r* = 0.377), urinary incontinence (*r* = 0.313), fecal incontinence (*r* = 0.226), constipation (*r* = 0.378), and poorer sexual function (*r* = − 0.182). Among the sociodemographic variables, only parity (*r* = 0.189) and BMI (*r* = 0.101) were correlated with depression.

**Table 2:** Pearson correlation coefficients between anthropometric, sociodemographic characteristics, lumbopelvic factors and depression during pregnancy

	Age (years)	BMI (kg/m2)	Income level (cordobas)	Parity	Low back pain (NPSR)	UI (ICIQ-SF)	FI (CCFI)	Constipation (WCSS)	Sexual Function (FSFI)
EDS	−0.084	0.101^*^	−0.070	0.189^**^	0.377^**^	0.313^**^	0.226^**^	0.378^**^	−0.182^**^
Age (years)		0.119^*^	0.199^**^	0.314^**^	−0.103^*^	0.003	−0.096	−0.005	−0.005
BMI (kg/m2)			0.047	0.154^**^	0.141^**^	0.177^**^	0.011	0.023	0.037
Income level (cordobas)				−0.089	−0.037	−0.020	−0.077	−0.021	0.086
Parity					0.153^**^	0.165^**^	−0.016	0.091	−0.028
Low back pain (NPSR)						0.286^**^	0.202^**^	0.441^**^	−0.120^*^
UI (ICIQ-SF)							0.204^**^	0.200^**^	−0.103^*^
FI (CCFI)								0.196^**^	−0.103^*^
Constipation (WCSS)									−0.200^**^

*Abbreviations*: *BMI* Body mass index, *CCFI* Cleveland Clinic Florida Fecal Incontinence, *EDS* Edinburgh Depression Scale, *FI* Fecal Incontinence, *FSFI* Female Sexual Function Index, *ICIQ_SF* International Consultation on Incontinence Questionnaire–Short Form, *NPRS* Numerical pain rating scale, *UI* Urinary Incontinence, *WCSS* Wexner Constipation Scoring System

^*^p < 0.05

^**^p < 0.001

**Fig. 2:**
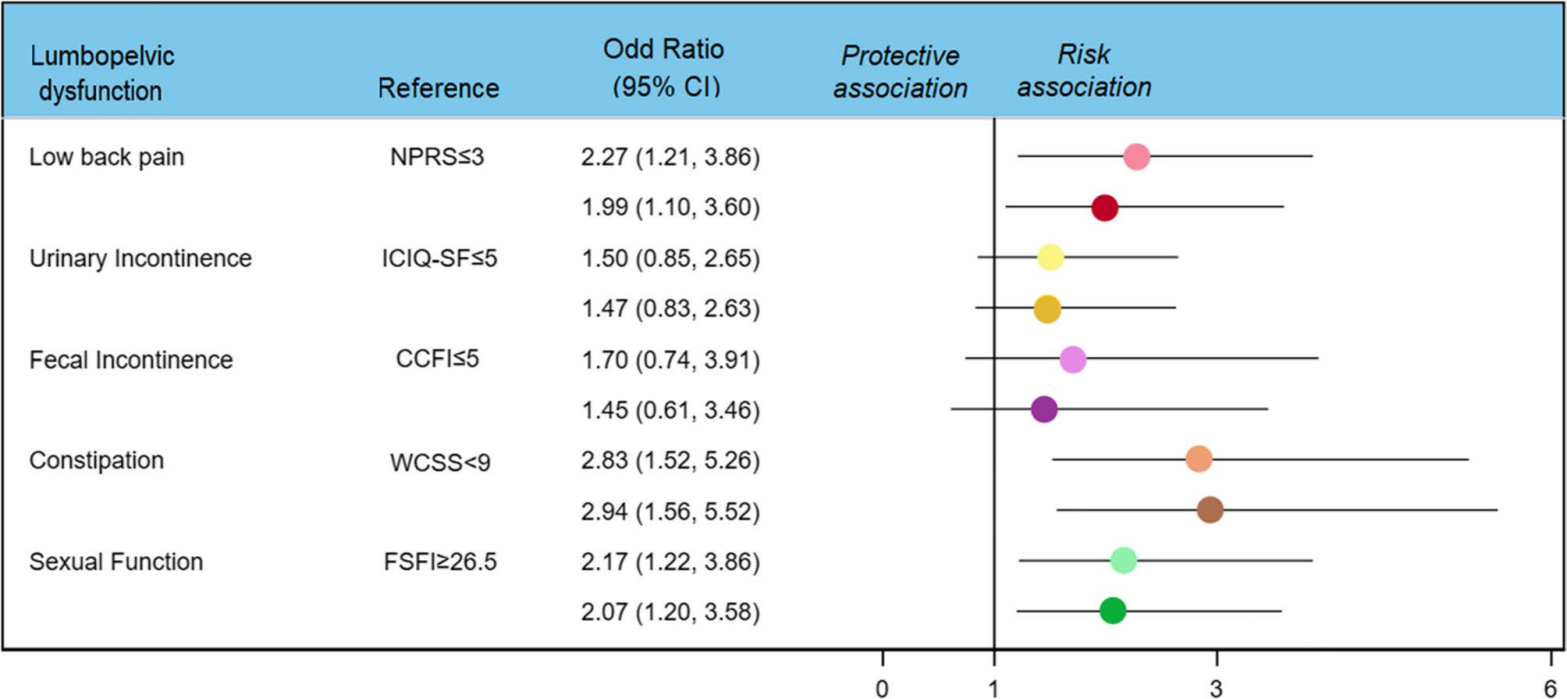
Crude and adjusted (age, BMI, family income level and parity) ORs from logistic regression models for the association between lumbopelvic dysfunction and depression during pregnancy. Depression condition assessed using a cut-off of ≥13 in the Edinburg Depression Scale. For each lumbopelvic dysfunction variable, light colors show unadjusted results and dark colors, adjusted results. Abbreviations: CCFI= Cleveland Clinic Florida Fecal Incontinence; EDS= Edinburgh Depression Scale; FSFI= Female Sexual Function Index; ICIQ_SF= International Consultation on Incontinence Questionnaire–Short Form; NPRS: Numerical pain rating scale; OR= Odd ratio; WCSS= Wexner Constipation Scoring System

According to crude logistic regression analyses (Fig. 2), the risk (OR; 95% CI) of depression was significantly elevated among women reporting low back pain (2.17; 1.22–3.86), constipation (2.83; 95% CI, 1.52–5.26), and sexual dysfunction (1.99; 1.17–3.40). However, urinary incontinence (OR, 1.50; 95% CI, 0.85–2.65) and fecal incontinence (1.70; 0.74–3.91) were not associated with depression. These associations persisted following adjustment for age, BMI, parity, and family income, as well as when the alternative depressive symptom threshold (EDS ≥ 11; Figure S1) was used.

## Discussion

Depression is a common condition during pregnancy and is influenced by a range of physical, mental, and sociodemographic risk factors. This study analysed data from 375 pregnant women to examine the role of lumbopelvic dysfunction as a risk factor for depression during this period. We found that low back pain, constipation, and sexual dysfunction were independently associated with depression even after adjusting for relevant sociodemographic and obstetric variables. Given that the study was conducted in a gynaecology and obstetrics service at a public hospital from Managua, Nicaragua, extrapolating the findings to other populations should be done with caution.

Overall, 22.4% of pregnant women in our sample presented symptoms of depression, a prevalence consistent with previous evidence [4], reinforcing the need for greater focus on maternal mental health, particularly to prevent postnatal depression. Early intervention may help prevent postpartum depression and improve outcomes for both mothers and children [43]. Among the lumbopelvic factors associated with depression in our population, significant differences were identified in symptoms such as low back pain and pelvic floor disorders (i.e., urinary incontinence, fecal incontinence, constipation, and/or sexual dysfunction).

Low back pain is one of the most common musculoskeletal complaints during pregnancy, largely due to hormonal, circulatory and mechanical changes that usually appears in the second trimester and has been associated with a negative impact on quality of life and psychological well-being [44]. In fact, up to 60% of pregnant women experience low back pain. Consistent with previous findings, our data show that those with moderate to severe pain have a greater risk of developing depressive symptoms [8].

In addition, during this period, there is biological plausibility for a relationship between low back pain and pelvic floor dysfunction. These conditions often coexist, potentially due to shared neuromuscular mechanisms [45]. The synergistic response between the abdominal and pelvic floor muscles suggests that low back pain may lead to poor pelvic stability and altered motor control of the pelvic floor muscles, particularly during increases in intra-abdominal pressure [46].

Pelvic floor disorders, including incontinence, constipation, and sexual dysfunction, are common and embarrassing problems associated with reduced quality of life and increased depressive symptoms [12]. However, it is traditionally associated with postpartum and older women [13, 47], our findings underline their relevance during pregnancy because pelvic floor disorders are associated with a 1.50 to 2.83-fold higher risk of depression symptoms in pregnant women, although only constipation (OR: 2.83) and sexual dysfunction (OR: 2.17) reached significance.

Our results align with previous evidence indicating that constipation is a common issue during pregnancy, particularly in the second trimester, that can negatively impact psychological well-being [48]. Factors such as reduced physical activity and poor dietary habits—both linked to constipation [49]—should be further explored in relation to depression, although variables such as income and education were already considered in our analysis. Sexual dysfunction, which is also common in pregnancy, may lead to decreased libido, desire, and orgasm, potentially affecting partner relationships [50]. While the association between sexual dysfunction and antenatal depression remains inconclusive [51], our results support the hypothesis that it may increase the risk of depressive symptoms. Moreover, the relationship between lumbopelvic dysfunction and depression may be bidirectional, as the neurochemical changes involved in depression could also impair lumbopelvic function [52].

The findings of this study have significant implications for health professionals and women. It highlights the co-occurrence of lumbopelvic dysfunction and depression during pregnancy, underlining the need for comprehensive treatments that consider the physical and emotional dimensions. Based on this association, therapeutic approaches should be adapted according to individual needs, as psychological support could help them to engage in treatments that could potentially prevent future complications.

This study has several limitations. First, its cross-sectional design prevents the establishment of causal relationships between depression and lumbopelvic dysfunction during pregnancy. Although our analyses identified significant associations, it remains unclear whether physical symptoms trigger the development of depressive symptoms or vice versa. This limitation should be considered when interpreting the results, and the findings should be considered with caution. Further longitudinal cohort studies or interventional designs are needed to confirm the directionality of these associations and to strengthen causal inference. Second, although we adjusted the analyses for some major potential confounders identified through the DAG procedures (i.e., age, BMI, parity, and income level), residual confounding (e.g., unmeasured psychosocial stressors, domestic violence, sleep quality, desired pregnancy or not) cannot be ruled out. Third, because the sample was limited to pregnant women for a gynecology and obstetrics service at a public hospital from Managua, extrapolation of the results to other sociodemographic and geographic settings should be performed with caution. Fourth, data collection through self-reports may introduce recall bias, although the questionnaires were administered face to face to minimize it. Fifth, our data are related to the second trimester of pregnancy, but further epidemiology studies are needed to assess the impact of lumbopelvic dysfunction on depression at different times in the perinatal period. Finally, pre-pregnancy baseline measurements of psychological variables were not taken into account for the analyses because, in our population, only 3% of women had a mental illness diagnosis before pregnancy; however, these data could be underestimated.

## Conclusion

This study provides further insight into the risk factors for depression during pregnancy, suggesting that women who experience low back pain, constipation, or sexual dysfunction may be more likely to present depressive symptoms. Therefore, prenatal care should incorporate effective screening and treatment strategies to prevent depression, including those related to lumbopelvic dysfunction. In addition, longitudinal studies aimed at assessing the long-term consequences of neglecting these factors for both mothers and babies are needed, as well as clinical trials evaluating the effectiveness of interventions aimed at jointly improving lumbopelvic dysfunction and mood disorders.

## Supplementary Information

Supplementary Material 1.

## Data Availability

Data is provided within the manuscript or supplementary information files.

## Abbreviations

BMI: Body mass index
CCFI: Cleveland Clinic Florida Fecal Incontinence
EDS: Edinburgh Depression Scale
FI: Fecal Incontinence
FSFI: Female Sexual Function Index
ICIQ-SF: International Consultation on Incontinence Questionnaire–Short Form
NPRS: Numerical pain rating scale
UI: Urinary Incontinence
WCSS: Wexner Constipation Scoring System
