# Supplementary material for: Lumbopelvic dysfunction and depression in pregnant women: a cross-sectional study
Source: BMC Pregnancy Childbirth. 2025 Nov 28;26:13. doi: 10.1186/s12884-025-08287-4 (PMC12764017; doi:10.1186/s12884-025-08287-4)
Supplement: Supplementary file 1 — Supplementary Material 1. [file 12884_2025_8287_MOESM1_ESM.docx]

**Supplementary material**

**Table S1.** STROBE Statement

|  | | **Item No** | **Recommendation** | **Page No** |  |
| --- | --- | --- | --- | --- | --- |
| **Title and abstract** | | 1 | (*a*) Indicate the study’s design with a commonly used term in the title or the abstract | 2 |  |
|  | |  | (*b*) Provide in the abstract an informative and balanced summary of what was done and what was found | 2 |  |
| **Introduction** | | | | |  |
| Background/rationale | | 2 | Explain the scientific background and rationale for the investigation being reported | 3, 4 |  |
| Objectives | | 3 | State specific objectives, including any prespecified hypotheses | 3,4 |  |
| **Methods** | | | | |  |
| Study design | | 4 | Present key elements of study design early in the paper | 4 |  |
| Setting | | 5 | Describe the setting, locations, and relevant dates, including periods of recruitment, exposure, follow- up, and data collection | 4 |  |
| Participants | | 6 | (*a*) Give the eligibility criteria, and the sources and methods of selection of participants | 4 |  |
| Variables | | 7 | Clearly define all outcomes, exposures, predictors, potential confounders, and effect modifiers. Give diagnostic criteria, if applicable | 5-7 |  |
| Data sources/ measurement | | 8 | For each variable of interest, give sources of data and details of methods of assessment (measurement). Describe comparability of assessment methods if there is more than one group | 5-7 |  |
| Bias | | 9 | Describe any efforts to address potential sources of bias | 7 |  |
| Study size | | 10 | Explain how the study size was arrived at | 7 |  |
| Quantitative variables | | 11 | Explain how quantitative variables were handled in the analyses. If applicable, describe which groupings were chosen and why | 5-7 |  |
| Statistical methods | | 12 | (*a*) Describe all statistical methods, including those used to control for confounding | 7-8 |  |
|  | |  | (*b*) Describe any methods used to examine subgroups and interactions | 8 |  |
|  | |  | (*c*) Explain how missing data were addressed | 8 |  |
|  | |  | (*d*) If applicable, describe analytical methods taking account of sampling strategy | NA |  |
| (*e*) Describe any sensitivity analyses | | | | 8 |  |
| **Results** | | | | | |
| Participants | | 13 | | (a) Report numbers of individuals at each stage of study—eg numbers potentially eligible, examined for eligibility, confirmed eligible, included in the study, completing follow-up, and analysed | 8, Table 1 |
|  | |  | | (b) Give reasons for non-participation at each stage | 8 |
|  | |  | | (c) Consider use of a flow diagram | NA |
| Descriptive data | | 14 | | (a) Give characteristics of study participants (eg demographic, clinical, social) and information on exposures and potential confounders | 8, Table 1, Table S1 |
|  | |  | | (b) Indicate number of participants with missing data for each variable of interest | 8 |
| Outcome data | | 15 | | Report numbers of outcome events or summary measures | 8,9 |
| Main results | | 16 | | (*a*) Give unadjusted estimates and, if applicable, confounder-adjusted estimates and their precision (eg, 95% confidence interval). Make clear which confounders were adjusted for and why they were included | 8,9, Table 2, Figure 1,2. Figure S1 |
|  | |  | | (*b*) Report category boundaries when continuous variables were categorized | Figure 1, Figure S1 |
|  | |  | | (*c*) If relevant, consider translating estimates of relative risk into absolute risk for a meaningful time period | NA |
| Other analyses | | 17 | | Report other analyses done—eg analyses of subgroups and interactions, and sensitivity analyses | 9 Table S2, Figure S1 |
| **Discussion** | | | | | |
| Key results | | 18 | | Summarise key results with reference to study objectives | 9-11 |
| Limitations | | 19 | | Discuss limitations of the study, taking into account sources of potential bias or imprecision. Discuss both direction and magnitude of any potential bias | 11 |
| Interpretation | | 20 | | Give a cautious overall interpretation of results considering objectives, limitations, multiplicity of analyses, results from similar studies, and other relevant evidence | 9-11 |
| Generalisability | | 21 | | Discuss the generalisability (external validity) of the study results | 9,11 |
| **Other information** | | | | | |
| Funding | | 22 | | Give the source of funding and the role of the funders for the present study and, if applicable, for the original study on which the present article is based | 12 |

NA: Not applicable

**Table S2.** Characteristics of the participants in the study

|  | Pregnant women without symptoms of depression (N:291) | Pregnant women with symptoms of depression (N:84) | p-value* |
| --- | --- | --- | --- |
| Age (years) | 30.11±4.79 | 29.52±4.59 | 0.260 |
| BMI (kg/m2) | 22.13±4.15 | 23.13±4.73 | 0.039 |
| Income level (cordobas) | 15657.30±9598.865 | 14184.85±8704.014 | 0.154 |
| Academic level, n(%) |  |  | 0.202 |
| Primary studies | 3 (0.8%) | 3 (0.8%) |  |
| Secundary studies | 53 (14.1%) | 33 (8.8%) |  |
| Technical/University | 199 (53.06%) | 84 (22.4%) |  |
| Parity, n (%) |  |  | 0.264 |
| Primiparous | 109 (29.06%) | 44 (11.73%) |  |
| Multiparous | 146 (38.93%) | 76 (20.26%) |  |
| History of mental illness, n(%) |  |  | 0.196 |
| No | 254 (67.73%) | 118 (31.46%) |  |
| Yes | 1 (0.26%) | 2 (0.53%) |  |
| EDS_Total Score | 4.40±3.43 | 15.20± 4.15 | <0.001 |
| Low back pain (NPSR) | 3.25±2.74 | 5.03±2.95 | <0.001 |
| UI (ICIQ-SF) | 2.41±3.49 | 4.83±4.77 | <0.001 |
| FI (CCFI) | 2.87± 2.77 | 4.07±3.26 | <0.001 |
| Constipation (WCSS) | 5.64± 4.02 | 8.45± 3.98 | <0.001 |
| Sexual Function (FSFI) | 25.98± 7.19 | 23.09±7.61 | <0.001 |

Values are means ± SD (quantitative variables) or n (%) (categorical variables). *Statistical significance p≤0.05

Abbreviations: BMI= Body Mass Index; CCFI= Cleveland Clinic Florida Fecal Incontinence; EDS= Edinburgh Depression Scale; FI= Fecal Incontinence; FSFI= Female Sexual Function Index; ICIQ-SF= International Consultation on Incontinence Questionnaire – Short Form; NPRS: Numerical pain rating scale; UI= Urinary Incontinence; WCSS= Wexner Constipation Scoring System.

**Figure S1**. Crude and adjusted (age, BMI, family income level and parity) ORs from logistic regression models for the association between lumbopelvic dysfunction and depression during pregnancy.


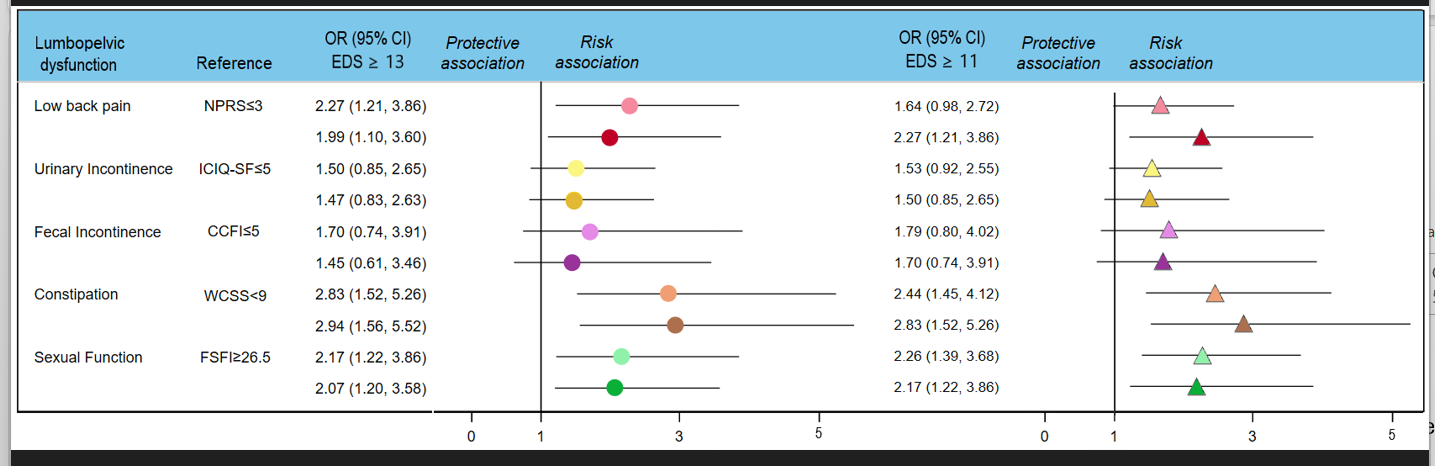


For each lumbopelvic dysfunction variable, light colors show unadjusted results and dark colors adjusted results. Abbreviations: CCFI= Cleveland Clinic Florida Fecal Incontinence; CI=confidence interval: EDS= Edinburgh Depression Scale; FSFI= Female Sexual Function Index; ICIQ_SF= International Consultation on Incontinence Questionnaire–Short Form; NPRS: Numerical pain rating scale; OR=Odd ratio; WCSS= Wexner Constipation Scoring System.
